# DJ-1 interactions with *α*-synuclein attenuate aggregation and cellular toxicity in models of Parkinson's disease

**DOI:** 10.1038/cddis.2014.307

**Published:** 2014-07-24

**Authors:** L Zondler, L Miller-Fleming, M Repici, S Gonçalves, S Tenreiro, R Rosado-Ramos, C Betzer, K R Straatman, P H Jensen, F Giorgini, T F Outeiro

**Affiliations:** 1 Department of NeuroDegeneration and Restorative Research, University Medical Center Göttingen, Göttingen, Germany; 2 Instituto de Medicina Molecular, Faculdade de Medicina da Universidade de Lisboa, Lisboa, Portugal; 3 Department of Genetics, University of Leicester, Leicester LE1 7RH, UK; 4 Danish Research Institute of Translational Neuroscience - Dandrite, Department of Biomedicine, Aarhus University, Aarhus, Denmark; 5 Centre for Core Biotechnology Services, University of Leicester, Leicester LE1 7RH, UK

## Abstract

Parkinson's disease (PD) is a devastating neurodegenerative disorder characterized by the loss of neurons in the substantia nigra pars compacta and the presence of Lewy bodies in surviving neurons. These intracellular protein inclusions are primarily composed of misfolded *α*-synuclein (aSyn), which has also been genetically linked to familial and sporadic forms of PD. DJ-1 is a small ubiquitously expressed protein implicated in several pathways associated with PD pathogenesis. Although mutations in the gene encoding DJ-1 lead to familial early-onset PD, the exact mechanisms responsible for its role in PD pathogenesis are still elusive. Previous work has found that DJ-1 – which has protein chaperone-like activity – modulates aSyn aggregation. Here, we investigated possible physical interactions between aSyn and DJ-1 and any consequent functional and pathological relevance. We found that DJ-1 interacts directly with aSyn monomers and oligomers *in vitro*, and that this also occurs in living cells. Notably, several PD-causing mutations in DJ-1 constrain this interaction. In addition, we found that overexpression of DJ-1 reduces aSyn dimerization, whereas mutant forms of DJ-1 impair this process. Finally, we found that human DJ-1 as well as yeast orthologs of DJ-1 reversed aSyn-dependent cellular toxicity in *Saccharomyces cerevisiae*. Taken together, these data suggest that direct interactions between DJ-1 and aSyn constitute the basis for a neuroprotective mechanism and that familial mutations in DJ-1 may contribute to PD by disrupting these interactions.

Parkinson's disease (PD) is a fatal neurodegenerative disease characterized by the loss of neurons in the substantia nigra pars compacta. Although most cases of PD are sporadic, several rare genetic forms of the disease have been identified that have contributed greatly to our understanding of the mechanisms underlying disease pathogenesis. Misfolded *α*-synuclein (aSyn) is the main protein component of Lewy bodies (LBs) – the primary pathological hallmark of PD^1^ – and aSyn has also been genetically linked to both familial and idiopathic forms of PD.^2, 3^ Misfolded aSyn protein assemblies range from monomers, oligomers and protofibrils, to fibrils and, finally, to inclusion bodies,^4^ with precursor oligomeric forms of aSyn apparently being more toxic than larger species.^5^ The physiological role of aSyn remains poorly understood, but it is thought to be associated with synaptic function/plasticity, cell differentiation and vesicular trafficking.^6^ aSyn dysfunction has been associated with many cellular effects, including proteasome impairment,^7^ disruption of endoplasmic reticulum-to-Golgi trafficking,^7, 8^ increased production of reactive oxygen species (ROS) and mitochondrial dysfunction.^9, 10^ On this evidence, several models of PD have been developed that overexpress wild-type or familial mutant versions of aSyn in organisms such as yeast, *C. elegans*, fruit flies and mice.^2^

Mutations in the *PARK7* gene account for ∼1–2% of early-onset recessive PD,^2, 11^ characterized by levodopa-responsive parkinsonism. *PARK7* encodes for DJ-1, a small conserved ubiquitously expressed protein of 189 residues primarily localized in the cytoplasm, but also found in the nucleus and associated with mitochondria.^12, 13, 14^ Crystallography has revealed that DJ-1 is a homodimer, which appears to be critical for its normal physiological function.^15, 16, 17^ A diverse set of potential functions have been suggested for DJ-1, including roles as an oxidative stress sensor (via a cysteine residue at position 106, C106), a protein chaperone, a protease, an RNA-binding protein, a transcription regulator, a regulator of mitochondria function and a regulator of autophagy.^18^ It is unclear which, if any, of these processes underlie DJ-1-dependent pathogenesis in PD.

Previous studies reported that DJ-1 can modulate the aggregation and toxicity of aSyn.^19, 20, 21^ Here, we investigated this relationship further and found that direct interactions between DJ-1 and monomeric/oligomeric aSyn are likely to underlie this process. Furthermore, we observed that familial PD mutations disrupt the interaction between DJ-1 and aSyn and abrogate the protective effects of DJ-1. In total, our work supports enhanced DJ-1 activity as an important target for therapeutic intervention in PD.

## Results

### DJ-1 binds monomeric and oligomeric aSyn *in vitro*

**Figure 1:**
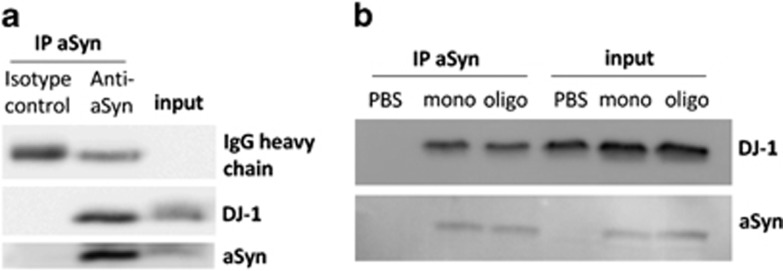
DJ-1 binds monomeric and oligomeric aSyn *in vitro*. (**a**) Co-immunoprecipitation of aSyn and DJ-1 in mouse brain homogenate demonstrates that DJ-1 and aSyn interact at endogenous levels. An isotype IgG control was performed to confirm the specificity of the interaction. One representative blot of three replicates is shown. (**b**) Co-immunoprecipitation of monomeric (mono) and oligomeric (oligo) aSyn with DJ-1 from lysates of DJ-1 overexpressing cells demonstrates that DJ-1 can bind both monomeric and oligomeric aSyn species *in vitro*. PBS instead of aSyn mixed with the lysate was used as negative control. The input lane is 2% of the sample prior to immunoprecipitation. One representative blot of three replicates is shown

Although previous studies found that DJ-1 can modulate aSyn aggregation and cytotoxicity,^22^ the nature of the possible physical interaction underlying this process is unknown. Thus, we sought to shed light into the interplay between the two proteins. To investigate whether an interaction between endogenous DJ-1 and aSyn is detected in the mouse brain, we generated C57BL/6 wild-type (WT) mouse brain homogenate and performed co-immunoprecipitation with anti-aSyn antibodies (Figure 1a). An interaction between DJ-1 and aSyn was detected indicating that DJ-1 and aSyn can specifically interact with each other at endogenous concentrations in the brain (Figure 1a).

Next, to determine if the interaction between DJ-1 and aSyn depends upon the aSyn aggregation species, we isolated purified aSyn monomers and oligomers as previously described,^23^ and incubated these preparations with extracts from HEK293 cells expressing myc-tagged DJ-1. Co-immunoprecipitation with anti-aSyn antibodies showed that both monomeric and oligomeric forms of aSyn interacted with DJ-1 with equivalent efficiencies (Figure 1b). These data indicate that DJ-1 physically interacts with soluble monomeric and oligomeric forms of aSyn *in vitro*.

### DJ-1 interacts with aSyn in living cells

**Figure 2:**
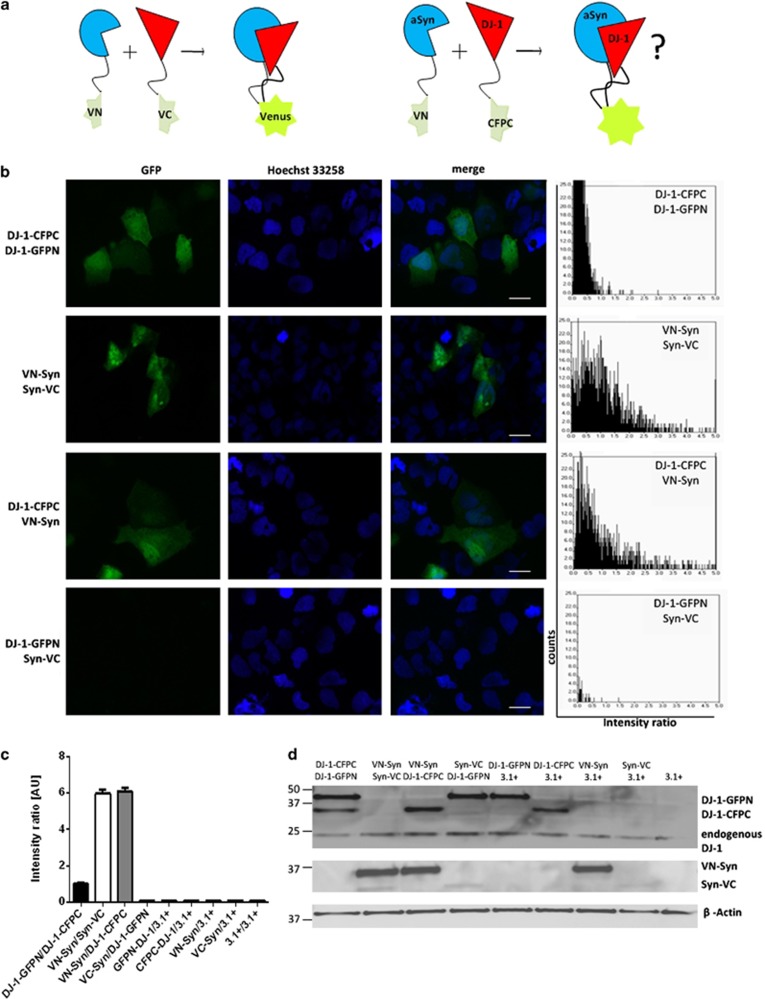
DJ-1 interacts with aSyn in living cells. (**a**) Schematic representation of the BiFC assay. The interaction of two proteins of interest (DJ-1 and aSyn) tagged with non-fluorescent fragments of fluorescent proteins (Venus or CFP) drives the reconstitution of the functional fluorophore resulting in fluorescence emission. (**b**) Representative sections showing the fluorescence complementation signal of DJ-1-GFPN/DJ-1-CFPC, VN-Syn/Syn-VC, VN-Syn/DJ-1-CFPC and Syn-VC/DJ-1-GFPN in living cells. Scale bar: 100 *μ*m. The distribution of ratios between GFP and mCherry emissions in individual cells co-transfected with plasmids encoding the proteins is shown in each graph. (**c**) The histogram shows the average ratio intensity for the indicated plasmid combinations (+S.E.M.). The combinations of the BiFC constructs DJ-1-GFPN/DJ-1-CFPC, VN-Syn/Syn-VC and VN-Syn/DJ-1-CFPC result in a fluorescence complementation signal indicated by positive intensity ratios when normalized to the mCherry transfection control. The same constructs combined with an empty vector control (3.1+) or transfection of the empty vector control alone do not result in positive fluorescence intensity ratios, indicating that fluorescence is only caused by the interaction of the two complementary constructs. (**d**) Immunoblotting analysis of the protein lysates obtained from transfected cell populations used in the BiFC experiment show the expression of the tested BiFC constructs, Upper panel: anti DJ-1 antibody, Middle panel: anti aSyn antibody, Lower panel: anti *β*-actin antibody

As DJ-1 can bind monomeric and oligomeric forms of aSyn *in vitro*, we next explored the interaction between the two proteins in living cells using bimolecular fluorescence complementation (BiFC), a powerful approach used extensively by our groups.^5, 17^ BiFC takes advantage of the reconstitution of non-fluorescent fragments of fluorescent proteins (GFP, RFP, Venus, etc.) to study protein–protein interactions in living cells.^24^ In this study, BiFC constructs encoding either DJ-1 or aSyn tagged with halves of GFP or Venus were co-transfected into HEK293 cells (Figure 2). aSyn dimerization can be preferentially detected using constructs in an antiparallel orientation in the BiFC assay^5^ with the N-terminal portion of GFP located at the N-terminus of aSyn and the C-terminal portion of GFP at the C-terminus of aSyn. Here, we utilized fusions of aSyn with fragments of Venus, because of the improved fluorescence emission of this protein compared with GFP.^25^ DJ-1 is known to form homodimers^16, 26^ and we previously characterized this dimerization in living cells using the BiFC assay.^17^ For the current study, we took advantage of the previously employed DJ-1 BiFC constructs, which encode DJ-1 tagged at its C-terminus with GFP or CFP halves (DJ-1-CFPC and DJ-1-GFPN; Figure 2). Fluorophore reconstitution has been shown to also occur for the combinations of Venus and GFP or CFP, respectively.^25^

To quantify the BiFC efficiency between DJ-1 and aSyn, we normalized the Venus/GFP emissions by the emissions of RFP or mCherry produced from a co-transfected expression plasmid. After subtraction of the background fluorescence, the intensity ratios between GFP and RFP fluorescence were calculated for every cell positive for red fluorescence, as previously described.^17^ Homodimeric interaction of DJ-1 or aSyn was confirmed by co-transfection of the two DJ-1 WT constructs and the two aSyn constructs, respectively (Figures 2b and c). Transfection of only one of the two BiFC constructs or the empty vector (pcDNA3.1+) did not lead to emission of green fluorescence (Figure 2c), whereas co-transfection of aSyn (VN-Syn) and DJ-1 (DJ-1-CFPC) BiFC constructs resulted in reconstitution of the fluorophores, indicating a direct interaction between these two proteins in living cells (Figures 2b and c). For the combination of Syn-VC and DJ-1-GFPN, no fluorophore reconstitution was observed (Figures 2b and c), indicating that the interaction between DJ-1 and aSyn can only be detected when the tags are present in a specific orientation, as previously observed for other proteins in BiFC experiments.^5^ Importantly, this result also confirms the specificity of the interaction, because the two fragments of the fluorescent proteins are present in the same cell but do not reconstitute the fluorophore if the orientation is not adequate. Expression of the DJ-1 and aSyn BiFC constructs was confirmed by immunoblotting for each experiment (Figure 2d). The reduced expression of Syn-VC construct compared to that of VN-Syn construct has been previously reported,^5^ and is likely because of the effects of the location of the tag on the protein. However, as shown for the VN-Syn/Syn-VC combination, this does not constrain the BiFC signal. Thus, our results indicate that DJ-1 and aSyn can indeed interact – likely directly, given the nature of the BiFC assay – in living cells.

### Familial PD mutations abrogate interactions between DJ-1 and aSyn

**Figure 3:**
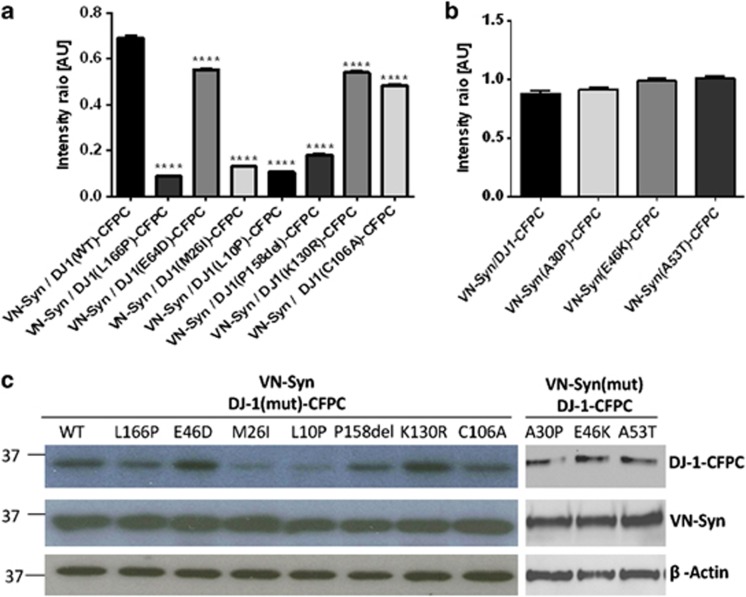
Familial PD DJ-1 mutations decrease DJ-1/aSyn interactions in living cells. (**a**) Effect of L166P, E64D, M26I, L10P, P158Δ, K130R and C106A DJ-1 mutations on the efficiency of fluorescence complementation between DJ-1 and aSyn BiFC constructs. The histogram shows the average ratio intensity (Green/Red) +S.E.M. per well. All the investigated DJ-1 mutations constrain the interaction efficiency of DJ-1 with aSyn significantly as indicated by decreased fluorescence intensity ratios compared to the DJ-1 (WT)/aSyn (WT) combination. ****P<0.0001. (**b**) Effect of A30P, E46K and A53T aSyn mutations on the efficiency of fluorescence complementation between DJ-1 and aSyn BiFC constructs. The histogram shows the average ratio intensity+S.E.M. per well. The ratio intensity of the BiFC signal from HEK293 cells co-transfected with VN-Syn (WT and mutants) and DJ-1(WT)-CFPC are not significantly different from one another, indicating that familial aSyn mutations (A30P, E46K, A53T) do not impair the interaction of aSyn with DJ-1. (**c**) Immunoblots of the lysates obtained from the transfected cell populations used in A. Upper panel: anti DJ-1 antibody, Middle panel: anti aSyn antibody, Lower panel: anti Tubulin (left panel); anti *β*-actin antibody (right panel)

To investigate whether the interaction between DJ-1 and aSyn was influenced by PD-causing mutations in one of the partners, HEK293 cells were co-transfected with combinations of WT VN-Syn and familial mutant DJ-1-CFPC constructs or familial mutant constructs of VN-Syn with WT DJ-1-CFPC, respectively. Quantification of the BiFC efficiency was performed as described above for each BiFC combination. The DJ-1 mutants L166P, M26I, L10P and P158Δ led to a dramatic reduction in the BiFC signal, thus indicating that the interaction with aSyn is abrogated compared to WT DJ-1 (*P*<0.0001; Figure 3a). The C106A, E64D and K130R mutations led to a slight but significant change in DJ-1/aSyn interactions (Figure 3a). Interestingly, the familial aSyn mutations A30P, E46K and A53T did not significantly alter the BiFC signal as compared to WT aSyn (Figure 3b).

Immunoblotting experiments showed that DJ-1 and aSyn BiFC constructs were highly expressed as WT proteins in HEK293-transfected cells, as were the mutant aSyn proteins (A30P, E46K, A53T) and the E64D, K130R and C106A DJ-1 mutants (Figure 3c). The L166P, M26I, L10P and P158Δ DJ-1 mutants were expressed at lower levels, which is consistent with previous findings showing that certain forms of mutant DJ-1 are less stable and more rapidly degraded than the WT protein.^17^ These data indicate that for this subset of DJ-1 familial mutations, the decrease in interactions with aSyn is due – at least in part – to decreased expression levels, which is likely representative of what occurs in patients. Nonetheless, these results indicate that familial mutations in DJ-1 can dramatically abrogate interactions with aSyn in living cells.

### DJ-1 modulates aSyn oligomerization

**Figure 4:**
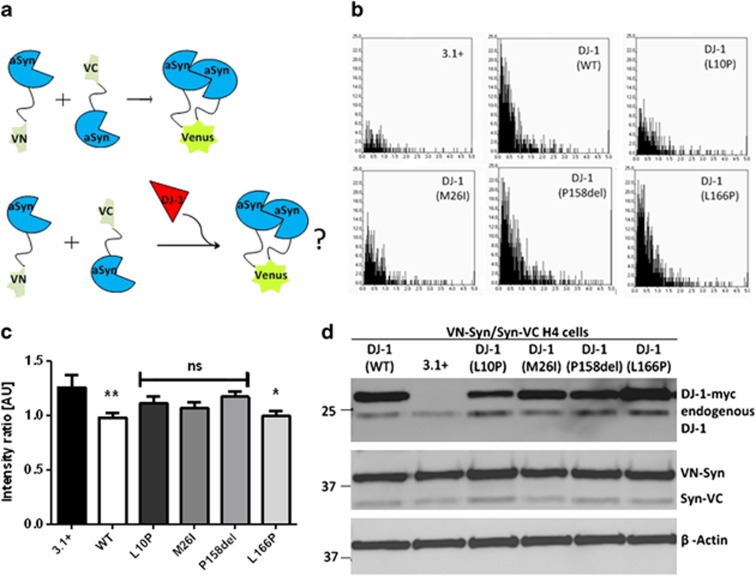
DJ-1 modulates aSyn oligomerization. (**a**) Schematic representation of the BiFC model of aSyn oligomerization used in this experiment. H4 cells stably expressing VN-Syn/Syn-VC were used to examine the impact of transiently transfected DJ-1 on aSyn oligomerization. (**b**) The impact of DJ-1 (WT and mutants, as indicated in the graphs) on aSyn oligomerization was examined. The distribution of intensity ratios between Venus and mCherry emissions in individual cells transiently transfected with the plasmid encoding myc-tagged DJ-1 (WT or mutant) or an empty vector control (3.1+) together with the mCherry control plasmid is shown in each graph. (**c**) The histogram shows the average ratio intensity+S.E.M. per well. A decrease was observed when VN-aSyn/aSyn-VC expressing H4 cells were transfected with DJ-1 (WT) compared to the empty vector control 3.1+ (*P*<0.01) indicating that WT DJ-1 interferes with aSyn oligomerization. DJ-1 mutants (L10P, M26I, P158Δ) failed to impair aSyn oligomerization to the same degree, while the L166P mutant significantly decreased the BiFC signal (*P*<0.05). (**d**) Immunoblot confirming the expression of transfected myc-DJ-1 constructs, as well as the stable expression of VN-Syn and Syn-VC. Upper panel: anti DJ-1 antibody, Middle panel: anti aSyn antibody, Lower panel: anti *β*-actin antibody

To investigate the impact of DJ-1 on aSyn oligomerization, WT and mutant (L10P, M26I, P158Δ, L166P) DJ-1-myc plasmids were transiently transfected, along with the mCherry transfection control plasmid, into H4 cells stably expressing VN-Syn and Syn-VC. This BiFC model of aSyn dimerization/oligomerization was previously described by our group and results in a clear fluorescence signal distributed throughout the cell^5^ (Figure 4a). When H4 VN-Syn/Syn-VC cells were transfected with myc-tagged WT DJ-1, a significant ∼22% decrease in the BiFC signal was observed in comparison to cells transfected with the empty vector control (pcDNA3.1+) (Figures 4b and c; *P*<0.01), indicating a decrease in aSyn oligomerization because of WT DJ-1. L10P, M26I and P158Δ mutant forms of DJ-1 failed to significantly impair aSyn oligomerization (Figures 4b and c), suggesting that these DJ-1 mutations lead to a loss of DJ-1 chaperone function. L166P mutant DJ-1, however, reduced the aSyn BiFC signal to a similar extent as WT DJ-1 (Figures 4b and c; *P*<0.05).

Expression of the transfected DJ-1 constructs was confirmed by immunoblotting (Figure 4d), supporting the finding that the familial mutations impair the capability of DJ-1 to antagonize aSyn dimerization/oligomerization. Interestingly, the M26I, P158Δ and L166P mutants were expressed at levels similar to WT DJ-1, which is in contrast to our immunoblotting data above as well as past studies that found decreased stability of these proteins.^17^ This is likely because of the N-terminal myc tag stabilizing these proteins, thereby permitting the direct testing of the effect these mutations have on aSyn oligomerization, independent of protein levels. In the case of L10P DJ-1, expression was lower than the WT DJ-1, which is consistent with previous studies.^17^

### DJ-1 reduces aSyn aggregation and cellular toxicity in yeast

**Figure 5:**
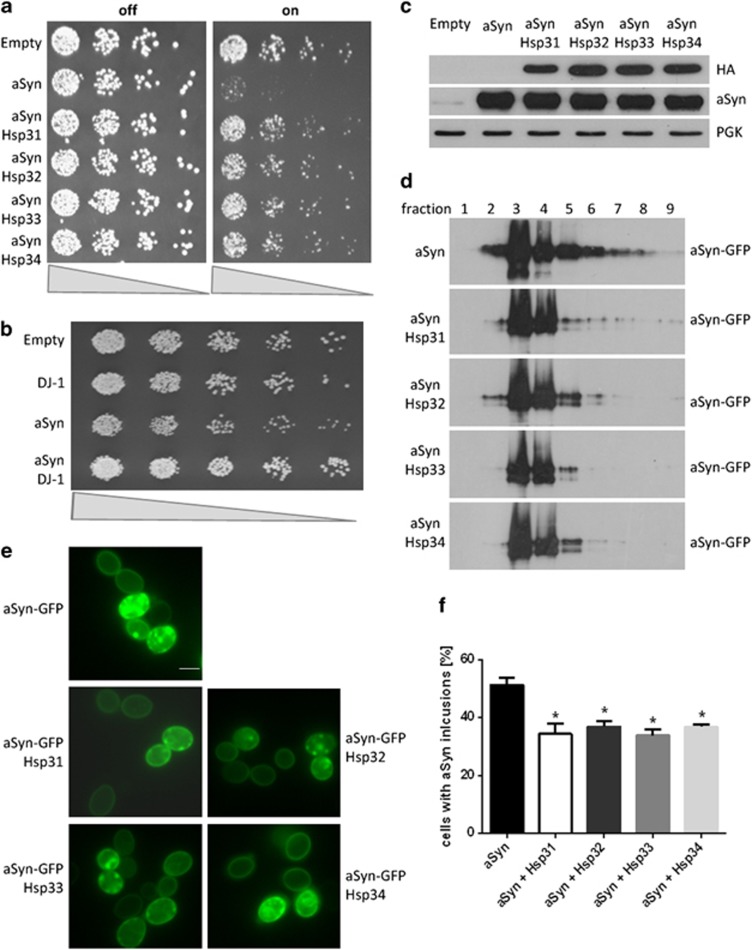
DJ-1 suppresses aSyn toxicity and aggregation in yeast. (**a**) Spotting assay of yeast cultures co-expressing aSyn and Hsp31, Hsp32, Hsp33 or Hsp34. Cultures were adjusted to OD_600 nm_=0.1, fivefold serially diluted and spotted onto media plates containing either glucose (aSyn and Hsps expression is repressed, ‘OFF') or galactose (expression is induced, ‘ON'). The spotting assay demonstrates that aSyn expression significantly reduces yeast growth and that co-expression of Hsp31, Hsp32, Hsp33 and Hsp34 reverses this toxicity. (**b**) Spotting assay of yeast cultures expressing aSyn and/or DJ-1. Cultures were adjusted to OD_600 nm_=0.1, fivefold serially diluted and spotted onto media plates to observe aSyn toxicity. This spotting assay confirms that aSyn expression significantly reduces yeast growth and that co-expression of human DJ-1 reverses the toxicity. (**c**) Immunoblotting demonstrates the expression levels of aSyn-GFP, Hsp31, Hsp32, Hsp33, and Hsp34 after 7 h of induction of aSyn expression. PGK was used as loading control. (**d**) Co-expression of aSyn with Hsp31, Hsp32, Hsp33 or Hsp34 reduces the high molecular weight aSyn species formed in yeast as analyzed by a sucrose velocity gradient and subsequent immunoblotting 7 h after induction of aSyn expression. (**e**) Representative images showing the intracellular localization of aSyn-GFP expressed alone or with Hsp31, Hsp32, Hsp33 or Hsp34. Scale bar: 5 *μ*m. (**f**) The histogram shows the mean percentage of yeast cells containing aSyn inclusions+S.E.M., assessed by fluorescence microscopy 7 h after induction of aSyn expression. Co-expression of Hsp31, Hsp32, Hsp33 or Hsp34 significantly decreases the number of aSyn inclusions (*P*<0.05)

Since the initial report on aSyn aggregation and toxicity in budding yeast,^9^ this organism has been extensively used to study the mechanisms underlying PD and other neurodegenerative disorders.^27, 28^ *S. cerevisiae* has four genes that belong to the DJ-1 superfamily (*HSP31, HSP32, HSP33, HSP34*).^29^ We found that co-expression of each of these four yeast genes reduced aSyn toxicity in yeast as indicated by spotting assays (Figure 5a). To investigate whether human DJ-1 has the same effect on aSyn toxicity, we repeated the experiment co-expressing human DJ-1 and aSyn in yeast. Concordantly, we found that aSyn toxicity is reduced by human DJ-1 (Figure 5b). Immunoblotting showed that the protein levels of aSyn did not change in the presence of the Hsps, demonstrating that the effect on toxicity was not simply because of decreased levels of aSyn in the cell (Figure 5c). Next, we performed a sucrose velocity gradient assay to separate proteins according to their molecular weight, as we previously described.^30^ Overexpression of all the DJ-1 superfamily members reduced the high molecular weight aSyn species (Figure 5d). This was further corroborated by counting the number of cells with aSyn inclusions by fluorescence microscopy (Figures 5e and f). We observed a ∼15% reduction in the number of cells with inclusions when the Hsps were co-expressed (*P*<0.01), which is in agreement with our data obtained in mammalian cells showing that DJ-1 modulates aSyn oligomerization.

## Discussion

aSyn plays an important role in the pathogenesis of several neurodegenerative diseases including PD and dementia with Lewy bodies.^31, 32, 33, 34^ Indeed, aggregated aSyn is the major component of Lewy bodies, protein inclusions that are found in the brains of PD and DLB patients.^31^ Moreover, mutations in or increased dosage of the aSyn-encoding gene lead to autosomal dominant familial PD. However, whether aSyn aggregation *per se* is cytotoxic and causes neurodegeneration, or the aggregation is an attempt to shield the neurons from toxic aSyn molecules of lower order, is still controversial.^3, 5, 35, 36, 37, 38^ DJ-1 is a ubiquitously expressed protein known to regulate the cellular response to oxidative stress by various mechanisms, including regulation of gene expression and mitochondrial integrity, induction of survival pathways and protein refolding.^12, 19, 22, 39, 40, 41, 42^ Mutations in the gene encoding DJ-1 can cause autosomal recessive PD,^1, 43^ although the underlying pathology leading to the development of PD is complex and various mechanisms are thought to be involved.^31, 44, 45^ Indeed, mitochondrial impairment and oxidative stress as well as protein misfolding are key events, but it is still not clear whether they are a cause or a consequence of the disease process.

DJ-1 has been found to be involved in these pathways in a neuroprotective manner. In line with our findings, DJ-1 has been shown to prevent protofibril formation by monomeric aSyn *in vitro*^19^ and reduce aSyn aggregation and toxicity.^22^ In addition, we also recently found that DJ-1 modulates mutant huntingtin aggregation *in vitro* and *in vivo*, in different models of Huntington's disease.^46^ In all of the reported studies, the chaperone-like activity of DJ-1 was dependent on the oxidative environment^46^ and, in particular, on the oxidation state of C106.^47^ Complementing these observations, our experiments find that the interaction of C106A mutant DJ-1 and aSyn is significantly impaired, further supporting the importance of the oxidation state of this residue for DJ-1 function and activation. Furthermore, DJ-1 has been found to bind copper ions at C106, which under critical conditions can be transferred to SOD1 (superoxide dismutase 1), thereby activating its catalytic function.^48^ This is thought to take place via a direct copper chaperone-like interaction between DJ-1 and SOD1.^49^

On the basis of the finding that DJ-1 prevents aSyn fibril formation,^19^ many reasoned that a direct interaction between the two proteins was likely to take place. However, previous attempts have failed to show a direct interaction between DJ-1 and aSyn,^50^ possibly because of the need of additional cofactors to stabilize the interaction. Therefore, our study demonstrates for the first time that DJ-1 and aSyn can directly interact *in vitro* and in living cells. As both the BiFC assay and our *in vitro* approaches demonstrate an interaction of DJ-1 with monomeric aSyn, our work is consistent with the idea that the DJ-1-aSyn interaction is an early event in the aSyn aggregation process.^19^

As mutations in the genes encoding these two proteins are implicated in familial PD,^1^ we hypothesized that these familial mutations could affect the interaction between the two proteins. Indeed, we demonstrate that familial PD mutations of DJ-1 affect this interaction. Furthermore, we show that DJ-1 reduces aSyn dimerization whereas familial PD mutant versions of DJ-1 fail to antagonize aSyn dimerization to the same degree. The mechanisms by which DJ-1 mutations cause the disease are not known, and an impairment of the interaction between DJ-1 and aSyn could provide a mechanistic explanation. Interestingly, the familial aSyn mutations interrogated in our study (A30P, E46K, A53T) did not abrogate interactions with WT DJ-1 by BiFC. To date, protein–protein interactions of aSyn have mostly been investigated in terms of aggregation (e.g. aggregation with tau or A*β*)^51, 52^ or membrane association,^53^ but not regarding potentially neuroprotective players in the PD context.

In contrast to the familial aSyn mutations, familial DJ-1 mutations – particularly L166P, M26I, L10P and P158Δ – were found to abrogate the interaction between DJ-1 and aSyn. These mutations impair DJ-1 homodimerization,^17, 54, 55^ suggesting that the functionality of the protein is perturbed. Moreover, these mutants increase the degradation and turnover rate of DJ-1,^17, 54, 55^ which explains the lower protein levels detected in these experiments. Our data suggest that the recessive familial mutations that impair dimerization and protein stability of DJ-1 also hinder the interaction between DJ-1 and aSyn, which is in agreement with several familial PD mutations affecting normal DJ-1 function. Notably, E64D DJ-1 is stable and dimerizes in a manner similar to WT^17^ and it is thus still unclear how this mutation might cause PD; our results show, however, that the E64D mutation restrains the interaction of DJ-1 with aSyn. The C106A mutation abolishes DJ-1 oxidation at this residue, which has been shown to be crucial for its chaperone activity.^47^ Therefore, our finding that the interaction of C106A DJ-1 with aSyn is significantly decreased compared to WT DJ-1 supports the hypothesis that DJ-1 acts as a chaperone on misfolded aSyn. As the K130R mutation – which prevents DJ-1 SUMOylation – also impairs the interaction with aSyn, the chaperone-like activity of DJ-1 may not only be dependent on oxidation but also upon SUMOylation.

As aSyn misfolds and aggregates in PD, and DJ-1 displays chaperone-like functions,^19, 46, 49^ we hypothesized that DJ-1 may modulate aSyn aggregation. Thus, we investigated the effect of DJ-1 on aSyn oligomerization using BiFC and observed that WT DJ-1 significantly reduces aSyn oligomerization in living cells. Our findings support previous studies that found DJ-1 prevented aSyn protofibril formation from monomeric aSyn^19^ and reduced aSyn aggregation.^47^ Taken together, these results suggest that DJ-1 may prevent aSyn aggregation by interfering with the initial steps in the aggregation process of di- or oligomerization. L10P, M26I and P158Δ DJ-1 are unable to decrease aSyn dimerization, clearly suggesting a loss of the chaperone DJ-1 function in these mutants. Unexpectedly, the L166P DJ-1 mutant significantly reduced aSyn oligomerization. Notably, the L166P protein is highly expressed in these experiments, likely as a result of protein stabilization because of the N-terminal myc tag. This suggests that this stabilized form of L166P is functional, and thereby is able to modulate aSyn aggregation.

Our findings in yeast, with human DJ-1 as well as the four yeast DJ-1 orthologs, further suggest that the chaperone function of DJ-1 is beneficial, as aSyn toxicity was ameliorated alongside with a reduction in aggregation.

In summary, we provide the first evidence that a direct interaction between aSyn and DJ-1 could be an important neuroprotective mechanism in the context of PD, and that the disruption of these interactions can contribute to the pathology caused by DJ-1 mutations. These observations support the notion that pharmacological interventions aimed at enhancing DJ-1 activity may provide a relevant therapeutic option in PD and in other synucleinopathies.

## Materials and Methods

### Immunoprecipitation of aSyn monomers and oligomers

HEK293 cells were transiently transfected by Neon (Invitrogen, Carlsbad, CA, USA) with myc-DJ-1 using 7.5 *μ*g DNA and 1100 mV for 20 ms, 2 pulses. After 24 h, the cells were trypsinized, collected by centrifugation, washed in Hank's buffered salt solution (HBSS) and lysed in RIPA buffer (50 mM Tris, 150 mM NaCl, 1% Triton X-100, 2 mM EDTA, 0.5% DOC, 0.1% SDS, Complete protease inhibitor cocktail mix, Roche, Basel, Switzerland). aSyn oligomers were produced by incubation of 10 mg/ml recombinant aSyn in phosphate-buffered saline (PBS) for 30 min on ice and isolated by gel filtration as previously described.^23^ 2 *μ*g of aSyn oligomers or monomers were mixed with 1.5 mg/ml HEK myc-DJ-1 lysate in PBS+0.5% TX-100 and allowed to interact over night at 4 °C with rotation. 2% of input sample was isolated prior to addition of 30 *μ*l CNBr-sepharose coupled to anti-aSyn (ASY-1), an affinity purified rabbit antibody against human aSyn as previously described.^56^ Samples were incubated at 4 °C with rotation for 2 h. Sepharose beads were isolated by centrifugation at 1500 r.p.m. for 1 min at 4 °C and washed five times in PBS+0.5% TX-100. Elution was obtained by adding non-reducing SDS-PAGE loading buffer (50 mM tris pH 6.8, 4% SDS, 40% glycerol, bromphenol blue) and incubated at 37 °C for 30 min followed by centrifugation. Diethyl ether (DTE) was added to the supernatant to the final concentration of 20 mM. Then the mixture was heated at 95 °C for 5 min and subsequently subjected to 10–16% SDS-PAGE and immunoblotted on a PVDF membrane (HYBOND LFP PVDF transfer membrane, GE Healthcare, Chalfont St Giles, UK; #RPN303LFP) using the primary antibodies rabbit anti-DJ-1 (1 : 2000, Abcam, Cambridge, UK; #ab18257) and rabbit anti-aSyn (1 : 1000, described above), as well as HRP conjugated secondary antibodies (1 : 1000, Dako, Glostrup, Denmark).

### Immunoprecipitation of endogenous aSyn and DJ-1

C57BL/6 WT mouse (6 months of age) brain homogenate was prepared in RIPA buffer. After retrieving the 10% input sample, the brain homogenate was incubated with 25 *μ*l CNBr-sepharose beads (GE Healthcare, #17-0430-01) coupled to anti-aSyn (ASY-1) or non-immune rabbit IgG (4 mg IgG/ml swelled sepharose) at 4 °C with rotation for two hours. Then, the beads were isolated, washed, eluted and immunoblotted as described above using the primary antibodies rabbit anti-DJ-1 (1 : 2000, Abcam #ab18257) and mouse anti-aSyn (1 : 1000, BD #610787, Franklin Lakes, NJ, USA) and polyclonal swine anti-rabbit IgG and polyclonal rabbit anti-mouse IgG HRP conjugated secondary antibodies (1 : 1000, Dako # P0217 and #P0260).

### Cell culture

HEK293 cells were cultured in DMEM high glucose, supplemented with 10% fetal calf serum (FCS), 100 units/ml penicillin and 100 *μ*g/ml streptomycin; H4 cells were grown in OPTI-MEM, supplemented with 10% FCS, 100 units/ml penicillin and 100 *μ*g/ml streptomycin. H4 cells stably transfected with two plasmids encoding aSyn tagged with either the N-terminal part or the C-terminal portion of Venus protein^5^ were cultured in OPTI-MEM with 30% FCS, 100 units/ml penicillin, 100 *μ*g/ml streptomycin and 0.1% G418. All cells were grown at 37 °C in 95% air / 5% CO_2_ atmosphere. For BiFC experiments, cells were grown on 6-well plates (1.5 × 10^5^ cells/well) pre-coated with 0.01% Poly-L-lysine solution or on 24-well plates at a density of 1 × 10^4^ cells/well 24 h prior to transfection.

### Generation of DNA constructs

The aSyn BiFC constructs (VN-Syn, Syn-VC, VN-Syn(A30P), VN-Syn (E46K), VN-Syn(A53T)) have been previously used in our laboratory,^5^ as well as the DJ-1 BiFC constructs (DJ-1-CFPC, DJ-1-GFPN, DJ-1(L10P)-CFPC, DJ-1(M26I)-CFPC, DJ-1(E64D)-CFPC, DJ-1(P158Δ)-CFPC, DJ-1(L166P)-CFPC)).^17^ The DJ-1(C106A)-CFPC and the DJ-1(K130R)-CFPC constructs were produced as described previously:^17^ the primer sequences are listed in the Supplementary Information; (Supplementary Table S1). To introduce the four different familial point mutations L10P, M26I, P158Δ and L166P into a DJ-1-myc plasmid vector (pGW1-CMV vector backbone), the QuickChange Site-Directed Mutagenesis kit (Stratagene, La Jolla, CA, USA) was employed according to manufacturer protocols using the primers listed in Supplementary Information; (Supplementary Table S2). All constructs were verified by DNA sequencing.

### BiFC assays

HEK293 cells were transfected with combinations of DJ-1 and aSyn-BiFC constructs using either Metafectene (Biontex, Munich, Germany) or Effectene (Qiagen, Venlo, Netherlands). Additionally, a transfection control plasmid encoding the red fluorescent mCherry protein or RFP was included in all experiments, allowing the selection of successfully transfected cells. The amount of the mCherry/RFP plasmid comprised one-fifth of the total DNA amount per well, whereas the BiFC constructs were used in equal amounts. The total DNA amount per well was 0.25 *μ*g in a 24-well plate format and 0.4 *μ*g in a 6-well plate format. To ensure optimal reconstitution of the fluorophores, particularly GFP, the transfected cells were incubated at 30 °C overnight prior to the image acquisition at the microscope. To explore the effect of DJ-1 on aSyn oligomerization, a BiFC-based aSyn dimerization/oligomerization model developed in our laboratory^5^ was used. H4 neuroglioma cells stably transfected with Venus-tagged aSyn BiFC constructs were transiently transfected with myc-DJ-1 plasmids using FuGene6 (Promega, Madison, WI, USA), including the same mCherry control plasmid as mentioned above. The total DNA amount per well was 0.5 *μ*g.

### Microscopy

Cells were imaged 24 h (H4 cells) or 48 h (HEK293 cells) post transfection with an Olympus XI81 system (Olympus Soft Imaging Solutions GmbH, Münster, Germany) or with an Olympus Sca
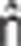
R screening station equipped with a 20 × LUCPlanFLN objective (NA=0.45) and a Hamamatsu ORCA-AG CCD camera (Hamamatsu Photomics, Hamamatsu, SZK, Japan). The light source was a MT-20 illumination system (Olympus Soft Imaging Solutions GmbH) with a high-stability 150 W xenon short arc burner. During imaging the cells were kept at 37 °C and 5% CO_2_. GFP/Venus BiFC fluorescence was detected using a 470/22 nm excitation filter and a 535/50 nm emission filter. mCherry/RFP expression was imaged using excitation at 556/20 nm and emission between 590 and 650 nm using the filter set 51019 (Chroma, Bellow Falls, VT, USA). In 24-well format experiments, 25 pictures were taken per well and about 800–1500 cells were identified to be mCherry positive for each condition in each independent experiment. The ratio between GFP/Venus and mCherry emission intensities was quantified for every cell expressing mCherry after subtraction of the background signal using the Sca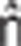;R analysis software as previously described.^17^ In 6-well format experiments, 100 pictures were taken per well. All experiments were performed in duplicate (6-well format) or triplicate (24-well format) and repeated three times. To obtain higher magnification representative images for each of the transfection combinations, transfected cells were fixed with 4% PFA in PBS (PAN) for 10 min at RT. After a washing step with PBS, cells were incubated for 10 min at RT in Hoechst 33258 (Molecular Probes, Carlsbad, CA, USA) solution for nuclear staining and then kept in PBS until used for microscopy. Representative images were taken with a Leica epifluorescence microscope (Leica, Wetzlar, Germany) using a 40 × objective.

### Immunoblotting of BiFC cell experiments

Directly after image acquisition at the microscope, cells were washed twice with sterile PBS and then lysed on ice for 10 min in lysis buffer as previously described^17^ or RIPA buffer (50 mM Tris, 150 mM NaCl, 1% Triton X-100, 2 mM EDTA, 0.5% DOC, 0.1% SDS, complete protease inhibitor cocktail mix, Roche). Lysates were centrifuged at 13 000 r.p.m. for 10 min at 4 °C. Supernatants were collected and protein concentration was determined by the Bradford method. Samples were stored at −80 °C until used. 10 to 30 *μ*g of protein per slot were separated on a 12% SDS polyacrylamide gel and transferred to a polvinylidene difluoride membrane. Membranes were incubated for 1 h in TBST (50 mM Tris, 150 mM NaCl, 0.05% Tween, pH 7.5) containing 5% milk to saturate all non-specific binding sites. Incubation with primary antibodies was overnight at 4 °C in 1% milk+TBST (1 : 1500 goat anti-DJ-1, Santa Cruz Biotechnology, Dallas, TX, USA; #Sc-27006; 1 : 3000 mouse anti-*α*-Synuclein, BD #610787; 1 : 5000 mouse anti-*β*-actin, Sigma-Aldrich, St. Louis, MO, USA; #A5441). Blots were developed using horseradish peroxidase (HRP)-conjugated secondary antibodies (anti-goat 1 : 10 000 Jackson Immunoresearch, West Grove, PA, USA; #705-035-003, anti-mouse 1 : 10 000 Sigma #50185-ILM-F) and the Immobilon chemiluminescent HRP substrate (Millipore, Billerica, MA, USA).

### Yeast plasmids, strains and culture conditions

Yeast work was performed in the wild-type BY4714 background (*MAT*, *ura3*Δ*0, leu2*Δ*0, his3*Δ*1, met15*Δ*0*). The yeast expression vector p426GAL was used to express human *SNCA* fused with GFP under a galactose-inducible promoter (*GAL1*) as previously described.^9^ Hsp31, Hsp32, Hsp33 or Hsp34 fused to a 6xHis-HA tag, were expressed under control of the *GAL1* promoter with BG1805 plasmids (Thermo Scientific, Waltham, MA, USA). To express the human DJ-1 gene under the regulation of the constitutive promoter *TPI*, the yeast vector pYX212 DJ-1 was used. Yeast cultures were transformed using a standard lithium acetate protocol. All strains were grown in synthetic complete medium without uracil and histidine (SC–URA-HIS), 6.7 g/l Yeast Nitrogen Base (BD), appropriate amino acid dropout mix (Sunrise Science Products, San Diego, CA, USA), 1% (w/v) raffinose or 1% (w/v) galactose. Yeast cells carrying the galactose-inducible aSyn-GFP construct alone or in combination with empty vector, Hsp31, Hsp32, Hsp33 or Hsp34 were pre-grown in SC-URA-HIS 1% raffinose for 24 h and then diluted back in order to reach an optical density (OD_600 nm_) of 0.4. For growth assays on solid medium, the cultures were serially diluted and spotted on SC-URA-HIS containing 1% galactose or 1% glucose (control) agar plates, which were then incubated at 30 °C for at least 48 h. For microscopy studies or protein extraction aSyn expression was induced in SC-URA-HIS 1% galactose for 7 h.

### Yeast protein extraction and immunoblotting

For total protein extraction, yeast cells were lysed in MURB buffer (50 mM sodium phosphate, 25 mM MES, pH 7.0, 1% SDS, 3 M urea, 0.5% 2-mercaptoethanol, 1 mM sodium azide) supplemented with protease and phosphatase inhibitor cocktail (Roche), with glass beads, three cycles of 30 s in the bead beater and 5 min on ice. Cell debris was removed by centrifugation at 10 000 *g* for 1 min at room temperature and after denaturation at 70 °C, the supernatant was collected. Equal volumes of total protein were analyzed by SDS-PAGE for the detection of aSyn levels. After electrophoresis, samples were transferred to a nitrocellulose membrane using a Trans-Blot Turbo transfer system (Bio-Rad, Hercules, CA, USA). Immunoblotting was performed following standard procedures with antibodies against aSyn (1 : 5000, BD #S63320), PGK (1 : 1000, Life Technologies Corporation, Carlsbad, CA, USA; #459250) and HA (1 : 1000, Santa Cruz Biotechnology #sc-7392).

### Sucrose gradient analysis of aggregation

Total protein from cells expressing WT or S129A aSyn was obtained and applied on a 5–30% sucrose gradient as previously described.^30^ Fractions were collected, precipitated for 4 h at 4 °C in trichloroacetic acid, washed in acetone three times and suspended in protein sample buffer (0.5 M Tris-HCl, pH 6.8, glycerol, 10% (w/v) SDS, 0.1% (w/v) bromophenol blue). Proteins were resolved by SDS-PAGE and estimation of the molecular sizes for each fraction were performed as previously described.^57^

### Yeast fluorescence microscopy

The percentage of cells with aSyn inclusions was determined by fluorescence microscopy using a Zeiss Axiovert 200 M (Carl Zeiss, Jena, Germany) wide-field fluorescence microscope equipped with a cooled CCD camera (Roper Scientific Coolsnap HQ, Martinsried, Germany) and a 63 × objective to acquire images containing at least 500 cells per strain, which were then counted using ImageJ. Yeast cells were grown as described above. After 7 h of induction, cells were collected by centrifugation and resuspended in PBS and 0.5% low melting agarose on a microscope slide.

### Statistical analyses

BiFC data were analyzed using the Mann–Whitney non-parametric test (GraphPad Prism 6), whereas yeast data were analyzed by the Tukey HSD (Honest Significant Difference) multiple comparison test (*α*=0.05). All data are reported as the mean± standard error of the mean (S.E.M.). Data were considered significant when *P*≤0.05. * indicates *P*≤0.05, ** indicates *P*≤0.01, *** indicates *P*≤0.001 and **** indicates *P*≤0.0001.

## Abbreviations

*μ*g: Microgram
aSyn: *α*-Synuclein
BiFC: Bimolecular fluorescence complementation
C: Celsius
*C. elegans*: *Caenorhabditis elegans*
CCD: Charge-coupled device
CFP: Cyan fluorescent protein
CNBr-Sepharose: Cyanogen bromide-activated Sepharose
CO_2_: Carbon dioxide
DLB: Dementia with Lewy bodies
DMEM: Dulbecco's modified Eagle's medium
DNA: Deoxyribonucleic acid
DOC: Deoxycholate
DTE: Diethyl ether
EDTA: Ethylenediaminetetraacetic acid
FCS: Fetal calf serum
GFP: Green fluorescent protein
HA: hemagglutinin
HBSS: Hank's buffered salt solution
HCl: Hydrochloric acid
HEK: Human embroynic kidney
His: Histidine
HRP: Horseradish peroxidase
HSD: Honest significant difference
HSP: Heat shock protein
LB: Lewy body
MAT: Mating type
MES: 2-(N-morpholino)ethanesulfonic acid
min: Minute
ml: Milliliter
mM: Millimolar
Mono: monomeric
NaCl: Sodium chloride
nm: Nanometer
OD: Optical density
Oligo: Oligomeric
PBS: Phosphate-buffered saline
PD: Parkinson's disease
PFA: Parafomaldehyde
PGK: Phosphoglycerate kinase
PVDF: Polyvinylidene fluoride
RFP: Red fluorescent protein
RIPA: Radio-immunoprecipitation assay
RNA: Ribonucleic acid
ROS: Reactive oxygen species
r.p.m.: Rounds per minute
RT: Room temperature
*S. cerevisiae*: *Saccharomyces cerevisiae*
SDS: Sodium dodecyl sulfate
SDS-PAGE: Sodium dodecyl sulfate polyacrylamide gel electrophoresis
S.E.M.: Standard error of the mean
SOD1: Superoxide dismutase 1
TBST: Tris buffered saline with Tween
W: Watt
WT: Wild-type
