# Supplementary material for: DJ-1 interactions with α-synuclein attenuate aggregation and cellular toxicity in models of Parkinson's disease
Source: Cell Death Dis. 2014 Jul 24;5(7):e1350–. doi: 10.1038/cddis.2014.307 (PMC4123098; doi:10.1038/cddis.2014.307)
Supplement: Supplementary Information [file cddis2014307x1.pdf]

Supplementary Information

WIPI-dependent autophagy during neutrophil differentiation of NB4 acute promyelocytic leukemia cells

Daniel Brigger[1,2], Tassula Proikas-Cezanne[3], and Mario P. Tschan[1,2,4]

[1]Division of Experimental Pathology, Institute of Pathology, and [2]Graduate School for Cellular and Biomedical Sciences, University of Bern, [3]Autophagy Laboratory, Interfaculty Institute for Cell Biology, Eberhard Karls University Tuebingen, Germany, and the [4]Department of Medical Oncology, Inselspital, Bern University Hospital, Bern, Switzerland.

Supplementary Figure 1. WIPI-2 levels in primary AML patient sample and NB4 APL cells upon ATRA treatment. (**a**) WIPI-2 mRNA levels of granulocytes from healthy donors and AML patient samples were measured using qPCR. The relative $^{\Delta}$Ct expression was calculated by the difference of WIPI-2 expression to the housekeeping genes HMBS and ABL. (**b**) mRNA levels of WIPI-2 were measured in control and ATRA treated NB4, NB4-R2 APL cell lines at day 4 qPCR. Values were normalized to the housekeeping gene HMBS and given as n-fold mRNA expression relative to the control of day 4. M.W.U, $^{*}p<0.05$.

Supplementary Figure 2. (**a**) Autophagy inhibitor LY294002 impaired ATRA-induced neutrophil differentiation. Neutrophil differentiation was assessed by measuring CD11b surface expression with FACS analysis at day 4 of ATRA treatment alone or in combination with 10µM LY294002. Bar graph of the median fluorescence intensity of 3 independent experiments is shown. M.W.U, $^{*}p<0.05$.

Supplementary Figure 3. Unaffected autophagy levels in shBECN1 knockdown cells upon short term ATRA treatment. (**a**) Quantification of endogenous LC3 puncta was assessed in SHC002 and shBECN1 APL cells upon ATRA and starvation treatment in combination with bafilomycin A1. Quantification was performed using ImageJ software. (**b**) The rate of long-lived protein degradation was measured in control, ATRA-treated or starved control cells or BECN1 knockdown cells, in the presence or absence of the lysosomal inhibitor bafilomycin A1. Values expressing the percentage of protein degradation are represented as mean of three independent determinations. (**c**) BECN1 Western blot analysis of NB4 SHC002 control and BECN1 knock down cells. Treatment as in b (BECN1 knockdown cells only treated with Baf A1 are missing). GAPDH was used as loading control. M.W.U, $^{*}p<0.05$, $^{**}p<0.01$.

Supplementary Figure 4. Autophagy flux measured in NB4 shPI3KC3 and shBECN1 knockdown cells. (**a**) The rate of long-lived protein degradation was measured in control and ATRA-treated SHC002 or WIPI-1 and WIPI-2 knockdown cells, in the presence or absence of the lysosomal inhibitor bafilomycin A1. Values, expressing the percentage of protein degradation, are represented as mean of three independent determinations. (**b**) The rate of long-lived protein degradation was measured in control and ATRA-treated SHC002 or PU.1 knockdown cells, in the presence or absence of the lysosomal inhibitor bafilomycin A1[21]. Analysis as in a. (**c**) NB4 parental and ATRA-resistant NB4-R2 cells were treated with 1µM ATRA for 4 days. Bafilomycin A1 was added after 3 days. Westernblot analysis of endogenous LC3-II protein. Three independently performed experiments were quantified. GAPDH was used as loading control. (**d**) Stable EGFP-Cherry-LC3 expressing NB4 cells were transduced either with SHC002, shPI3KC3 or shBECN1 and were treated with 1µM ATRA for 4 days. Bafilomycin A1 was added after 3 days. Representative pictures of EGFP-Cherry-LC3 puncta from 3 independently performed experiments are shown. (**e**) Long-lived protein degradation in ATRA-treated SHC002 control or PI3KC3 and BECN1 knockdown cells. Analysis as in a. (**f**) AnnexinV/PI FACS staining upon ATRA treatment was measured in SHC002, shWIPI-1, shPI3KC3 and shBECN1 APL cells. M.W.U, $^{*}p<0.05$, $^{**}p<0.01$.

Suppl. Figure 1

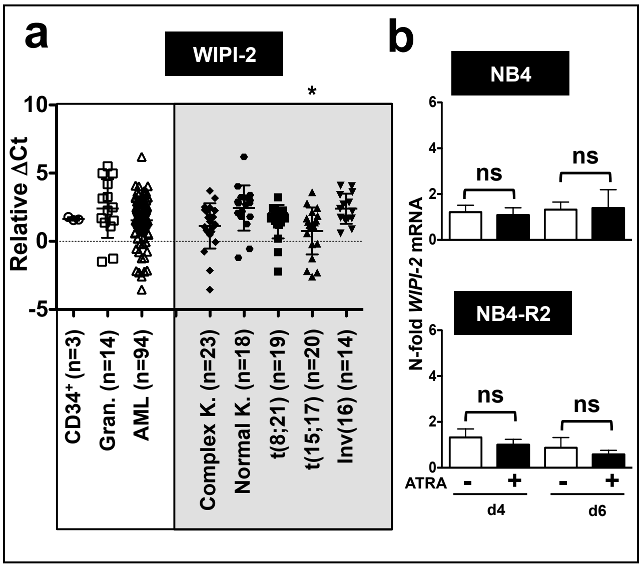

Suppl. Figure 2

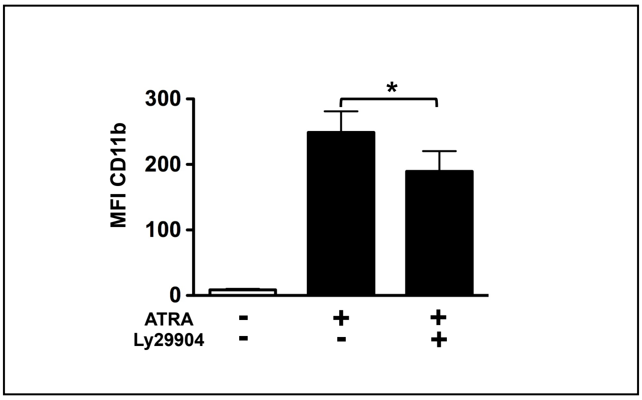

Suppl. Figure 3

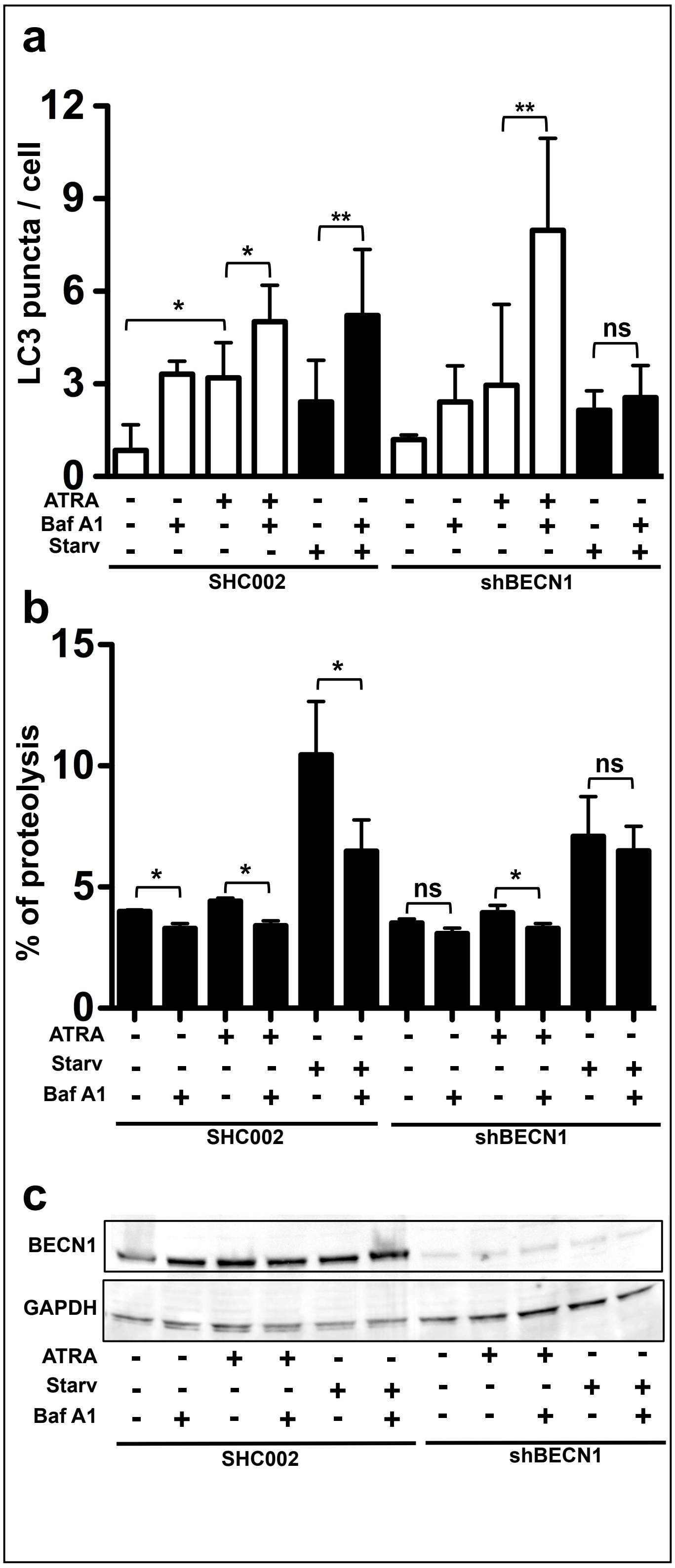

Suppl. Figure 4

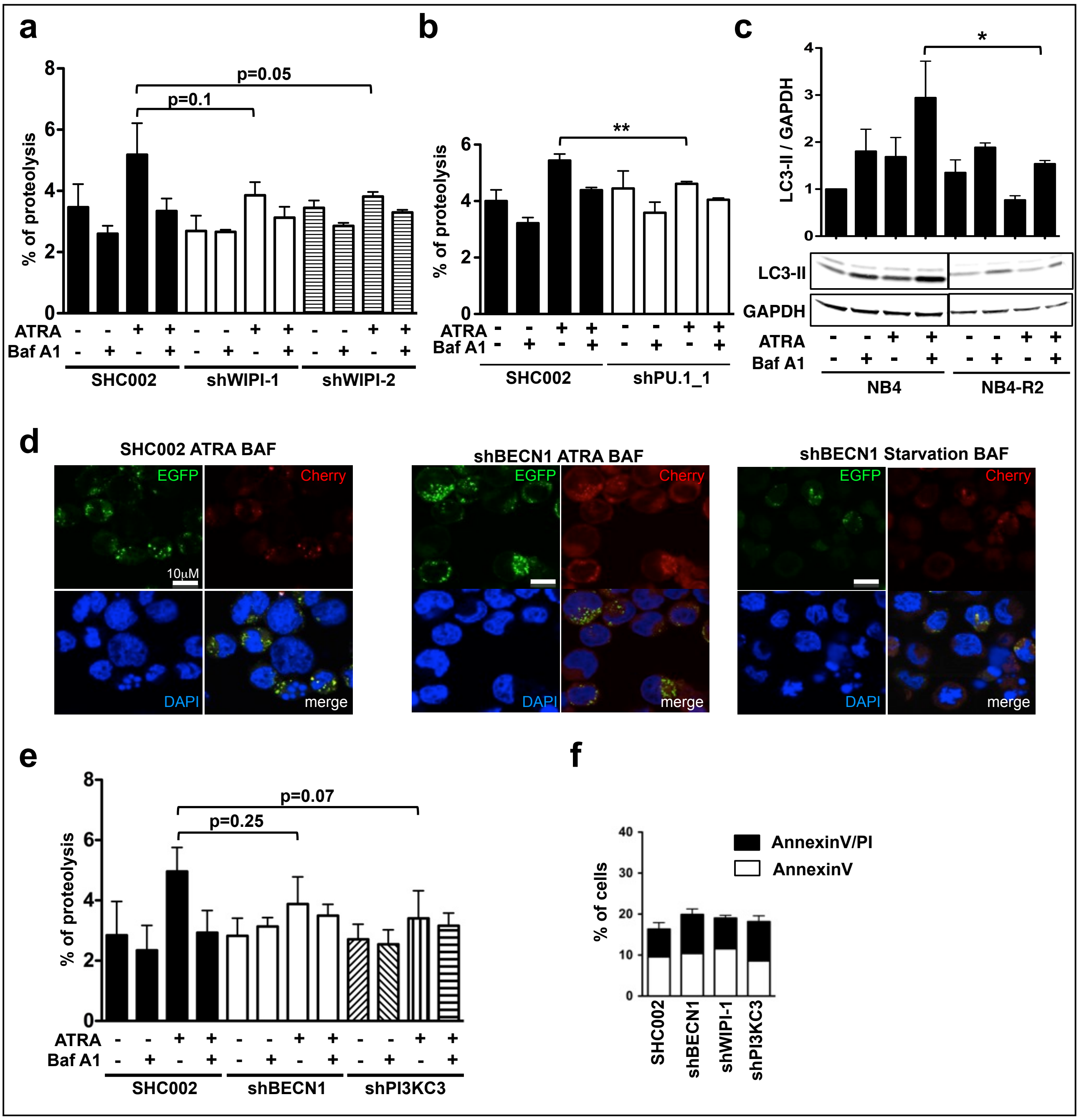

Supplementary Table 1. AML patient characteristics from the HOVON/SAKK cohort.

			Patient characteristics			Cytogenetics				
	**Cohort**	**Variables**	Age (y)	Sex (female/male)	**Total**	inv (16)	t(8;21)	t(15;17)	CK	NK
WIPI-1	HOVON/ SAKK	**Range**	15-74							
		**Mean/median or %**	43.89/43.00 (mean/median)		**100%**	16.3	20.4	20.4	24.5	18.4
		**No. of patients**	98		**98**	16	20	20	24	18

			Patient characteristics			Cytogenetics				
	**Cohort**	**Variables**	Age (y)	Sex (female/male)	**Total**	inv (16)	t(8;21)	t(15;17)	CK	NK
WIPI-2	HOVON/ SAKK	**Range**	15-74							
		**Mean/median or %**	43.02/43.00 (mean/median)		**100%**	14.9	20.2	21.3	24.5	19.1
		**No. of patients**	94		**94**	14	19	20	23	18

FAB, French-American-British; CK, complex karyotype; NK, normal karyotype
